# What our children lost and gained at the time of school closure during the Covid-19 pandemic: a study on psychological distress, behavioural concerns and protective factors of resilience among preschool children in Kerala, India

**DOI:** 10.1186/s12939-023-02090-3

**Published:** 2024-01-23

**Authors:** Jose Vincent, Resmi Madhusoodanan Santhakumari, Anjana Nalinakumari Kesavan Nair, Anisha Sharahudeen, Asvini K.P, Meenu Maheswari Suresh, Mathew J. Valamparampil, Gayathri A.V, Chintha Sujatha, Anish Thekkumkara Surendran

**Affiliations:** 1 Department of Community Medicine, Amala Institute of Medical Sciences, Thrissur, Kerala India; 2 District Hospital, Kerala 689641 Kozhencherry, India; 3 Government Medical College Thiruvananthapuram, Thiruvananthapuram, Kerala 695011 India; 4 PHC Kadachira, Kannur, Kerala 670621 India; 5 Sree Chitra Tirunal Institute for Medical Sciences and Technology, Trivandrum, 695011 India; 6 WHO medical consultant, NTEP, Kerala Ernakulam, India

**Keywords:** COVID 19, Early childhood development, DECA P2, Behavioural Concerns

## Abstract

**Background:**

The pandemic has put at risk the social and emotional development of children on account of the paucity of arenas for social interaction. This study from Kerala, India was conducted to assess the resilience factors, behavioural concerns, psychological distress symptoms among the children aged 3 to 5 years. We also tried to look into the lost opportunities that could have aided the social and emotional development of children like peer interaction, child care hours.

**Methods:**

The cross-sectional study was conducted among the children aged 3 to 5 years. A total of 535 children attending the immunisation clinics were enrolled by consecutive sampling. Devereux Early Childhood Assessment P2 (DECA P2) questionnaire was used to assess the levels of resilient factors and behavioural concerns in the study population.

**Results:**

We observed a high proportion of children in the area of need category of protective factors under DECA P2. The proportion of children falling under area of concern was 64.5%, 49%, 68.4% for attachment/relationship, self-regulation, and initiative respectively. 24.9% study subjects have a behavioural concern score that puts them in the area of need category. The logistic regression model we created identified ‘Male Gender,’ ‘Mothers could spend only less time for child care’ and ‘electronic devices used as pacifier’ as significant predictors for belonging to Area of need Behavioural Concerns T score category.

**Conclusion:**

A large proportion of children aged between 3 to 5 years with reported behavioural concerns and lack of protective factors for socioemotional development. This can be attributed partly to the ongoing pandemic and its associated restrictions. The increased child care hours invested by parents or grandparents could have sized down the full impact that the pandemic would have had on the socio emotional development of the child. Increased time spent using electronic devices coupled with dwindled opportunities for interaction with peers have been notable challenges.

**Supplementary Information:**

The online version contains supplementary material available at 10.1186/s12939-023-02090-3.

## Introduction

COVID 19 pandemic has caused major disruption in the life of children all around the world [1]. The closure of schools, the shift of academic activities to online platforms, the paucity of opportunities to interact with other children are new realities the children had to face on account of the pandemic [2]. The schools were closed in the state of Kerala on 10 march 2020 [3]. The academic activities were completely shifted to online platforms and through television channels. The schools remained closed till October 2021.

Early childhood education and care (ECEC), defined as any regulated arrangement that provides education and care for children from birth to the start of primary education [4]. In the Indian context Anganwadis (most peripheral grass root level centre for mother and childcare centre in India), preschools and kindergartens contribute towards imparting ECEC for children aged 3 to 6 years. The preschools, kindergartens play a great role in building the physical, social and psychological domains of resilience [5].

The quality of preschool education also has an important role in early development. Children who have high-quality early childhood education experiences have better cognitive and socio-emotional outcomes [6].

Preschools and kindergartens offer opportunities for peer interaction and children build build emotional contacts outside the family [7]. Interaction with peers at school (for example child-initiated play) has an influence on behaviour, cognition, and personality. School functions as a physical space where children share values, interests, thoughts, hopes and emotions among peers [3, 5, 8]. The closure of schools and the absence of social gatherings have greatly curtailed the opportunities available for peer interaction.

The child does not experience the pandemic in vacuum. She/he experiences it in the company of people closely linked to her/his life like parents, siblings, other family members, friends, and peers [9]. The events occurring in these ‘linked lives’ have important influence on the way the child experiences the pandemic. The parents and other care providers could experience parenting stress on account of the economic uncertainties and lack of social support system [10]. For those who had to work from home, boundaries between work and family life are blurred [11]. A stressful collision of roles as a parent, a spouse, an employee, an employer, a caregiver, etc., might be experienced by parents [10]. The time available for the parent for childcare activities might have changed on account of the pandemic. The interaction with the linked lives can serve as a source of support or strain for the child [9]. Confinement and crowded conditions, due to the pandemic related movement restrictions, have been associated with large increases in domestic violence [12]. Reduced opportunities for social interaction and engaging in physical activity can impact the development of the child [13].

The children had to rely on mobile phones, tablets, and personal computers for academic activities as they were carried out via online platforms [13]. Mobile phones and internet usage are recognised as potential objects of non-substance addiction [14]. Academic failures, physical and mental health problems, sleep disturbances are associated with excessive, uncontrolled, or inappropriate use of the smartphone [15].

Symptom manifestations of stress, anxiety, and post-traumatic stress disorder have been observed among children during the pandemic [16, 17]. Resilience is defined as the adaptive capacity available at a given time in a given context that can be drawn upon to respond to current or future challenges facing the individual, through many different processes and connections [18]. Social-level systems (families, friends), community-level systems (schools, emergency service systems, etc.), and macrosystems (such as government-level systems) have profound influence on building resilience in children. The age group 0 to 6 is a crucial period in the development of positive traits like initiative, self-regulation, and attachment [18].

Disrupted access to health, education and social services worsen health outcomes in disadvantaged children. Longer term impacts on health, well-being, literacy, income, professional opportunity, housing, and intergenerational effects are possibly overwhelming [19].

The pandemic presents many risk factors that can derail the development trajectories of children. Many opportunities that have aided children’s development are lost. These can have long term and short-term impact on the social and emotional development of the child. Children could have been the segment of the population that to bear an unevenly severe impact of the pandemic. The effect of the pandemic on children is intangible and very often overlooked; at times contributing gross inequity in bearing the burden of the pandemic. The pandemic can very well be treated as a child rights and equity crisis [19]. The present study from the state of Kerala, India during COVID 19 pandemic analyse the psychological distress faced by children aged between 3 to 5 years and the protective factors (resilience) and behavioural concerns experienced by them.

## Methods

### Study design, setting and methods

We conducted a cross sectional study in the Indian state of Kerala during July-December 2021. Study collected information on children aged 3 to 5 years. We included children from hospitals of five districts of the state to ensure geographical representativeness. The children attending the immunisation clinics of two primary level hospitals, two secondary level hospitals and two tertiary level hospitals were part of the study. Thus, all layers of the three-tier health system, both public and private could be represented. Five districts were randomly selected from the total 14 districts in the state and hospitals were conveniently selected to be part of the study. The tools used for the study were pertaining to pre-school children, age three to five years. Immunisation clinics of the selected hospitals were the study setting because apparently healthy children in this age group usually visit them to receive vitamin A prophylaxis as a part of National Blindness Control Program. The investigators were present on each of the study sites. The accompanying parents of the eligible children were invited to participate in the study by the investigators. Consenting parents were required to fill the questionnaire at the time of visit (offline) or they were sent it online to be filled at their convenience. The site investigator administered the tool directly under circumstances when participants found it difficult to fill the questionnaire on their own. We promoted the self-administration of questionnaire through online mode because of the prevailing social distancing protocols. In built explainers and guidance were provided to those filling self-administered questionnaire to ensure data quality. Such a mixed approach ensured data from children belonging to various occupation and socio-cultural groups as well.

We decided to enrol around 500 children to the study. Consecutive sampling was adopted for recruiting the study participants till adequate sample size was achieved. Parents who were unwilling to participate, children with known development delays were excluded from the study. We arbitrarily decided to enroll around 500 children to the study. A study done by Niu et al. in China, which investigated the developmental characteristics of resilience in children aged 3–5, employed a similar sample size of 570 [20]. Consecutive sampling was adopted for recruiting the study participants till the required sample size was achieved. We monitored the number of children enrolled from different sites daily and stopped the data collection at the end of the day when the overall sample size crossed 500. The data collection occurred from November 2021 to January 2021.

### Study tool and variables

Devereux Early Childhood Assessment Clinical Form, pre-school (DECA-P2) is a tool with high reliability and validity, used to assess the social and emotional development of the children. DECA P2 contains a 38-item questionnaire (27 strength-based protective factor items and 11 items on the Behavioural Concerns scale) intended for children aged 3 to 5 years [21]. Resilience/protective factors could be assed in three domains- “Attachment/ Relationship,” “Initiative” and “Self-regulation.” Behavioural concerns constituted the fourth domain. Each item had 5 responses namely never, rarely, occasionally, frequently, very frequently; with an associated score of 0,1,2,3,4 respectively. DECA P2 questionnaire yield raw scores for each child under four domains. The raw scores are standardized to corresponding T score for each domain using the individual child profile chart available with DECA P2. T scores for the protective domains; Attachment/ Relationship, Initiative and Self-regulation were categorised as “Strength” (T Score more than or equal to 60), “Typical” (T score between 41 and 59) and “Area of need” (T score of 40 and below) [22]. These domain score under “Attachment/ Relationship,” “Initiative” and “Self-regulation” were added up to obtain raw score for total protective factors (TPF) [23]. TPF was also categorised similar to its component domains to assess the overall protective factors after determining the corresponding T scores from the chart. T scores for the fourth domain, behavioural concern could be categorised into two namely; “Area of need” (T score 60 or more) and “Typical” (T score less than 60) categories. Low T Score for the protective domains and high T Score for the ‘behavioural concern domain’ indicated lower resilience. T scores that fell in to the “Area of need” category indicated the need for an intervention. The tool for the current study was filled by one of the parents of the child based on the child’s behaviour in past 4 weeks. Malayalam translation of the original tool was done by two bilingual experts independently and a final draft was prepared based on consensus. The draft was finetuned after back translation by another bilingual expert who was not aware of the content of the original tool. The tool was implemented in a small number of parents (*n* = 10) to check for any ambiguity before the commencement of its use.

Apart from the DECA-P2 tool, another questionnaire enquiring the socio-economic details, pandemic related behaviours, interaction of children and their family members, use of electronic gadgets was also used for the present study.

We asked the responding parent to report any change in the average amount of time the father, mother, or the grandparents spend with the child after the onset of the pandemic. If they reported an increase or decrease in the average amount of time, they were asked to quantify it in minutes per day. The total gain in time and loss in time spent on childcare was calculated by adding up the duration of time reported. Average amount of time child gained or lost was also calculated.

### Statistical analysis

The statistical analysis was done using SPSS 23.0 (SPSS Inc. Released 2007. SPSS for Windows, Trial Version 16.0. Chicago, SPSS Inc). Quantitative variables like age and quality time with the child are expressed as mean with standard deviation. Categorical variables were expressed as frequencies and percentages. Responses of items in DECA P2 questionnaire were converted to domain specific T scores based on standard charts [23]. Statistical significance of associations was tested using chi-square test for categorical exposure factors and odds ratio with confidence intervals were estimated for all exposure variables. Analysis was done using Multiple logistic regression model to identify the independent predictors for the children being in the risky category of ‘Area of need’ for ‘total protective factors T score’ and ‘behavioural concern T score.’

### Ethics

Ethical clearance for the study proposal has been obtained from the Institutional Human Ethics Committee (IHEC) of Sree Uthradam Thirunal academy of medical sciences (IHEC No: 233 dated 3/11/2021). Informed written consent was obtained from the parents. The study complied with the institutional and national research committee ethical standards, and with the 1964 Helsinki Declaration and subsequent amendments. Data safety and confidentiality was ensured throughout the conduct of the study.

## Results

A total of 535 children were part of the study. As shown in Table 1, 126 (23.6%) children had a completed age of 3 years; 198 (37%) had 4 years and 211 (39.4%) had 5 years. Proportion of boys were 46.7% (*n* = 250) and that of girls were 53.3% (*n* = 285). The age of father ranged between 24 to 52 years with mean (SD) 36.6 (4.3) years. The mean (SD) age of the mother was 31.5 (3.78) years with min- max of 22—48 years. Most of the parents (70.7% of fathers and 85.1% of mothers) in our study were graduates.

**Table 1:** Baseline characteristics of the study participants

Variable	Category	Frequency	Percentage
Gender	Male	250	46.7
	Female	285	53.3
Age in completed years	3 years	126	23.6
	4 years	198	37
	5 years	211	39.4
Education Level of the Father	SSLC/10th Standard Equivalent	157	29.3
	Degree	240	44.9
	Post-Graduation & Above	138	25.8
Education Level of the mother	SSLC/10th Standard Equivalent	80	15
	Degree	248	46.4
	Post-Graduation & Above	207	38.7

For 46.5% fathers (*n* = 249,46.5%) there was no change in the time spent on child care during the pandemic. While 150 (28%) participants reported that the father could only spent lesser time for child care and 136 (25.4%) male parents could dedicate more hours to child care. On an average there was a minimal loss of 48.52 h of paternal spending of time for the entire cohort of 535 children, amounting to a daily loss of 5.4 min per child. Similarly, maternal time spent on child care was not affected by pandemic in 225 (42%) cases. The pandemic provided more opportunities for the mother to spend time for child care in case of another 225 (42%), but 80 (15%) could invest only lesser time compared to the pre-pandemic period. Overall, the cohort was gaining 762.1 maternal hours per day, accounting for a gain of 85.5 min per day per child. The cohort of 535 children gained 746.6 additional hours per day from their grandparents during the pandemic, translating to 83.7 min per child per day. However, the quality time lost by the children was from their friends and the cohort experienced a loss of 288.7 h per day, equivalent to 32.4 min per day per child. One third of the cohort (*n* = 171, 32%) were not having any opportunities to mingle with children other than their siblings. In case of 261 children (48.8%), either the mother or the father was working from home during the pandemic.

Table 2 summarises parents’ perception on children’ attitude towards schooling and the use of electronic gadgets by the child. The question on reopening of physical pre-school sessions/kindergarten was not applicable to 86 respondents as their children had never been to any of these institutions. Of the remaining 449 participants 269(59.9%) responded that their children ‘often’ asked about school reopening; 94(20.9%) asked about school reopening ‘very often’; 48(10.7%) ‘rarely’ asked about school reopening and 38(8.5%) ‘never’ asked about it. Two-third of the eligible children (*n* = 295, 65.7%) children had ‘often’ expressed feelings of missing their schoolmates; 70 (15.6%) ‘very often’ expressed such feelings. The number of children who ‘rarely’ missed their schoolmates/ friends at school was 59(13.1%) and 25(5.6%) children ‘never’ expressed such feelings. The number children who were able to play with other children (other than their siblings) during the pandemic days was 171(32%). The average time the child spends watching television/using computer or mobile phone per day was found to be 3.52 (1.2) hours with a minimum of 2 and maximum of 12. Forty-six (8.6%) children had a computer device/mobile phone designated for their use only. The activities of the children on devices like mobile phones, tablets and laptops were never monitored or rarely monitored by 24(4.5%) and 27 (5%) respondents respectively. The number of parents who admitted to using mobile phone/tablet/computer as pacifier ‘very often’ and ‘often’ were 20 (3.7%) and 297 (55.51%) respectively.

**Table 2:** Child’s attitude towards schooling and use of mobile phones at the time of pandemic

Variable	Category	Frequency	Percentage
The frequency at which the child asks about the Re opening of School/Kindergarten(*n* = 449)	Very often	94	20.94
	Often	269	59.91
	Rarely	48	10.69
	Never	38	7.10
The frequency at which the child talks about missing friends/peers(*n* = 449)	Very often	70	15.59
	Often	295	65.70
	Rarely	59	13.14
	Never	25	5.57
Parent’s rating of the interaction of the child with other children while meeting them in person during the pandemic (*n* = 535)	Excellent	130	24.3
	Good	276	51.6
	Average	93	17.4
	Below Average	32	6
	Poor	4	0.7
Parent’s rating of the interaction of the child with other children while meeting them during online meetings. (*n* = 535)	Excellent	54	10.09
	Good	256	47.85
	Average	162	30.28
	Below Average	47	8.79
	Poor	16	3
The frequency at which the activities of the child on mobile phones/ tablets /computers are monitored by parents. (*n* = 535)	Very often	300	56.1
	Often	184	34.39
	Rarely	27	5
	Never	24	4.5
The frequency at which mobile phones/ electronic devices are used to pacify the child. (*n* = 535)	Very Often	20	3.7
	Often	297	55.51
	Rarely	119	22.2
	Never	99	18.5
The child has a mobile device/tablet/computer device of his own		46	8.6

Only 215(40.2%) among the total 535 children had any of the psychological symptoms.

320 (59.8%) did not have any psychological symptoms. Of the 535 study participants 48 (9%) children had difficulties in sleeping; 38(7.1%) showed withdrawn or aggressive behaviour; 45(8.45%) children were afraid to be left alone. Clinging or dependent behaviour was the most common psychological distress symptom and it was present in 83(15.5%) children. There were 20 children (3.7%) who complained of nightmares; 27(5%) children had manifestation of new fears; 22(4.1%) children had shown decreased interest in playing and engaging in playful activities; 35(6.5%) children had history of being sad, crying more than usual or for no apparent reason.

Distribution of four domains of protective factors and the behavioural concerns measured by DECA P2 questionnaire is given in Table 3. We noticed that majority of children fell in the risky category of ‘area of need’ in all protective domains (attachment/relationship-64.5%, self-regulation-49%, initiative-68.4% and total protective factors-68%). But majority (402, 75.1%) of the children included in the study had a ‘typical’ T score for behavioural concerns and 133(24.9%) children had T scores that categorise them as the risky group ‘area of need’.

**Table 3:** Classification of study population based on the T scores obtained from DECA P2 questionnaire (*n* = 535)

Domain	Category	Frequency	Percentage
Protective factor-Attachment/relationship	Area of need	345	64.5
	Typical	172	32.1
	Strength	18	3.4
Protective factor-Self-Regulation	Area of need	262	49
	Typical	245	45.8
	Strength	28	5.2
Protective factor-Initiative	Area of need	366	68.4
	Typical	158	29.5
	Strength	11	2.1
Total protective factors	Area of need	364	68
	Typical	157	29.3
	Strength	14	2.6
Behavioural Concerns	Area of need	133	24.9
	Typical score	402	75.1

Factors associated with T score indicating whether the child is in ‘area of need’ or not were assessed separately for ‘behavioural concerns’ and ‘total protective factors. The total protective factor T score categories of ‘typical’ and ‘strength’ were combined to create binary variable. Male gender, usage of electronic devices as pacifiers and ‘mother could only spend lesser amount of time for child care during the pandemic’ were found to be factors associated to ‘area of need’ T score for behavioural concerns. Usage of electronic devices (mobile phones /tablets personal computers) as pacifiers, father, mother or grandparents could only spent lesser time for child care pandemic showed association with getting a ‘area of need’ total protective factors (Table 3).

Multiple logistic regression models were created with behavioural concern T score category (‘area of need’ category or not) and total protective factors T score (‘area of need’ category or not) as outcome variables. Male Gender (AOR,1.82(1.24–2.8), *p* = 0.003), mothers could spend only less time on child care (AOR, 1.9(1.08–3.19) *p* = 0.03), and electronic devices used as a pacifier (AOR, 2.16(1.4–3.40) *p* = 0.001), were significant predictors for belonging to Area of need Behavioural Concerns T score category. Electronic devices used as pacifiers (AOR, 1.84(1.27–2.71) *p* = 0.001) and ‘explained the pandemic situation to the child’ (AOR, 0.35(0.14–0.84), *p* = 0.02) were the predictors for belonging to the ‘Area of need’ total protective factors T score category. It should be noted that explaining the pandemic situation to the child is a protective factor (Supplementary Tables 1 and 2).

## Discussion

We observed a high proportion of children in the area of need category of protective factors under DECA P2. The proportion of children falling under area of concern was 64.5%, 49%, 68.4% for attachment/relationship, self-regulation and initiative respectively. The protective factors are resources, processes, or characteristics that help an individual buffer risk and build resilience. The paucity of such protective factors underscores the importance of strengthening societal -level (such as families and friends), community-level (such as schools), and macrosystems-level (such as civil society) mechanisms. The support system the society, school and family provides can help build the protective factors that enable the child to tide over adverse events in future and help him/her to achieve his full potential [24]. COVID 19 pandemic might have caused or aggravated the problem of children not developing protective factors during the early part of their childhood. With regard to behavioural concerns, 24.9% of the children had scores that put them in the ‘area of need’ category. This almost translates into every fourth child having a behavioural concern. Children, especially the young ones, are also in a position of vulnerability during the pandemic. Social connection is crucial for identity and wellbeing at young ages. Studies exploring the impact of COVID 19 pandemic on children have identified emotional reactivity of children can be increased, emotion regulation can be decreased [25]. Existing mental health problem make the children more vulnerable to the increase in emotional reactivity. Particularly vulnerable are children with existing mental health problems. Data from previous epidemics demonstrate that children who experienced isolation measures were more inclined to experience PTSD [26].

The logistic regression model identified that children of ‘mothers could spend less time with the child during the pandemic’ were more prone to fall in to ‘Area of need’ Behavioural Concerns T score category. Allowance to work from home should naturally give the parents more time to interact with child and could have a mediating effect in bringing down the effect of isolation the child is experiencing. But the ability of the parents to allocate time for child care, the financial situation of the parents, parents’ ability to cope with stress [27], the occurrence of incidents of domestic violence [28], the methods employed to engage the children and the alternative methods employed to ensure social connection of children can complicate the picture [29]. The stressors experienced by parents can lead onto abuse and maltreatment of children [27]. Excessive use of electronic devices can contribute to poor social and emotional development of children. With conventional arenas of peer interaction and group play not available, the increased reliance on screen time for entertainment can result in sleep deprivation, poor mental and physical health [30, 31]. Even though majority of the parents in the study claimed to monitor the activities of children on electronic devices, the quantity and variety of content available on such devices can be a parenting challenge. Boys appears to have less resilience according to the multivariate model. Informing the child about the pandemic situation showed a protective effect. Tang et al. (2021) also reports that open parent–child discussion about the pandemic situation helped the older children and adolescents to tide over the psychological crisis [32]. Having an open conversation about the COVID 19 situation provides an opportunity to be empathetic with the child. Allowing the child to air his concerns about the pandemic is also a supportive behaviour [33]. Our study also found that keeping the children informed during the pandemic has a beneficiary effect even in younger children.

The prevalence of psychosocial distress symptoms among the study population is alarming- 15.5% children exhibited clinging behaviour; 9% had difficulties in sleeping. This finding sits right with similar studies done during the pandemic which have reported an increased prevalence of psychosocial distress symptoms, dependant behaviour among children who are subject to social confinement. A 10.5% prevalence of psychological distress symptoms was found in a study cross-sectional study done in Guangdong province, China. High prevalence of psychological distress in quarantined children and adolescents has also been observed [34]. The study conducted in India found quarantined children experienced helplessness (66.11%), worry (68.59%) and fear (61.98%), compared to non-quarantined children [35].

Our study found out that majority of the parents claiming that the time spent on child care was not overly affected by the pandemic. We have identified a loss in overall child care hours for male parents. An overall decrease in time spent on peer interaction was also noted. This could have placed an increase demand on the mother as evidenced by the overall increase in the time spent by the mothers on childcare. In situations when the mother could not invest time in childcare an associated area of need T score was obtained for the child for behavioural concerns as indicated by our regression model. Alternate modalities of child care have kicked in as evidenced by the increase in the child care hours of the grandparents. As expected, time spent for interaction with other children has dwindled. The time and space that out to have been occupied by social activities and group play could have been replaced by the use of electronic devices as shown by the increase in time spent by children on such devices. In a study done in Turkey among children aged between 7 and 13, 41.5% of the parents stated that their child gained weight and an increased tendency to sleep and use internet more frequently was also observed [1].

COVID 19 pandemic and the disruption it caused provided a unique opportunity to explore how it might have impacted the normal development of children. The study could be timed perfectly so that the lived experience of the caretakers could be captured. We included almost 500 children in the study that too from 5 of the total 14 districts in Kerala. This might have enabled the study to capture a pan Kerala picture. The study also helped to identify children as a vulnerable group when it comes to the effects of pandemic. Further research should address the inequity issue so that better coping strategies can be employed.

### Limitations

The pandemic and associated restrictions were universal. It ruled out the possibility of having control group. The outcomes reported in our study may not be attributed solely to the pandemic. The study employed an arbitrarily chosen sample size. Due to the ongoing pandemic restrictions we had to resort to a non-random sampling technique. This must have caused selection bias. The differential rate of entry from different districts and levels of hospital could also have contributed to selection bias. The study captured the pandemic experience of children as narrated by parents. It could have caused reporting bias.

## Conclusions

A large proportion of children aged between 3 to 5 years with reported behavioural concerns and lack of protective factors for socioemotional development. This can be attributed partly to the ongoing pandemic and its associated restrictions. The increased child care hours invested by parents or grandparents could have sized down the full impact that the pandemic would have had on the socio emotional development of the child. Increased time spent using electronic devices coupled with dwindled opportunities for interaction with peers have been notable challenges. However, explaining the pandemic situation to the child and reduced usage of phone as a pacifier are protective behaviours that can build resilience of the child. It becomes obvious that children have to bear an unequal share of burden as far as the impact of the pandemic is concerned. The inequity of this scenario needs urgent redressal.

The arbitrarily chosen sample size and the non-random sampling technique employed could have affected the generalisability of our conclusions. But the conclusions from our study sample strongly suggest that future pandemic response should consider the uneven burden borne by children. Institutional mechanisms should be in place to negate the psychological distress faced by children during pandemics. Policies should be in place to negate the effect that the pandemic had on the children. Right, actionable information should made be accessible to all stakeholders involved in childcare.

### Supplementary Information

**Additional file 1: Supplementary table 1. **Factors associated with area of need scores for Behavioural Concerns. **Supplementary table 2. **Factors associated with area of need scores for Total Protective Score.

## Data Availability

The data set will be available on request.
