# Supplementary material for: What our children lost and gained at the time of school closure during the Covid-19 pandemic: a study on psychological distress, behavioural concerns and protective factors of resilience among preschool children in Kerala, India
Source: Int J Equity Health. 2024 Jan 23;23:14. doi: 10.1186/s12939-023-02090-3 (PMC10807164; doi:10.1186/s12939-023-02090-3)
Supplement: Supplementary file 1 — Additional file 1: Supplementary table 1. Factors associated with area of need scores for Behavioural Concerns. Supplementary table 2. Factors associated with area of need scores for Total Protective Score. [file 12939_2023_2090_MOESM1_ESM.docx]

Supplementary tables

| **Supplementary table 1**  **Factors associated with area of need scores for Behavioural Concerns** | | | | |
| --- | --- | --- | --- | --- |
|  | Area of need scores for behavioural concern | Typical Scores | P value | Odds ratio |
| Male Gender | 76(30.4%) | 174(69.6%) | <0.01^#^ | 1.75(1.18-2.6) |
| Was allowed to work from home | 56(21.5%) | 205(78.5%) | 0.08 | 0.7(0.47-1.04) |
| Explained the pandemic situation to the child | 118(23.9%) | 375(76.1%) | 0.09 | 0.57(0.29-1.1) |
| Child could play with children from another house holds | 50(29.2%) | 121(79.8%) | 0.11 | 1.4(0.93-2.11) |
| Child owns a mobile phone/tablet/pc | 16(34.8%) | 30(65.2%) | .10 | 1.7(0.89-3.22) |
| Has used mobile phones /tablets personal computers to pacify the child | 96(30.3%) | 221(69.7%) | <0.01^#^ | 2.13(1.39-3.26) |
| Monitors child’s activity on phones /tablets personal computers | 116(24%) | 368(76%) | 0.17 | 0.63(0.34-1.17) |
| Children whose mothers could only spent less time for child care | 29(36.3) | 51(63.8) | 0.01^#^ | 1.19(1.16-3.18) |
| Children whose fathers could only spent less time for child care | 45(30) | 105(70) | 0.09 | 1.45(0.95-2.21) |
| Children whose grandparents could only spent less time for child care | 19(35.2) | 35(64.8) | .07 | 1.175(0.96-3.17) |
| Children who could only spent less time for interaction with other children | 71(28.2) | 181(71.8) | .09 | 1.4(0.94-2.07) |
| **Supplementary table 2**  **Factors associated with area of need scores for Total Protective Score** | | | | |
| Male Gender | 167(66.8%) | 68(33.2) | 0.57 | 0.90 (.63-1.29) |
| Was allowed to work from home | 181(69.3%) | 80(30.7%) | 0.53 | 1.13(.78- 1.62) |
| Explained the pandemic situation to the child | 328(66.5%) | 165(33.5%) | 0.01 | 0.33(0.13- 0.80) |
| Child could play with children from another house holds | 124(72.5%) | 47(27.5%) | 0.13 | 1.36(0 .91- 2.03) |
| Child owns a mobile phone/tablet/pc | 36(78.3%) | 10(21.7%) | 0.12 | 1.77(0.86 - 3.65) |
| Has used mobile phones /tablets pcs to pacify the child | 234(73.8%) | 83(26.2%) | 0.001^#^ | 1.91(1.32-2.76) |
| Monitors child’s activity on phones /tablets personal computers | 327 (67.6) | 157 32.4 | 0.53 | .78(0.41-1.5) |
| Children whose mothers could only spent less time for child care | 63 (78.8) | 17(21.3) | 0.03^#^ | 1.89(1.07-3.35) |
| Children whose fathers could only spent less time for child care | 114 (76) | 36 (24) | 0.02^#^ | 1.71(1.13-2.63) |
| Children whose grandparents could only spent less time for child care | 44 (81.5) | 10(18.5) | 0.03^#^ | 2.21(1.09-4.51) |
| Children who could only spent less time for interaction with other children | 162(64.3) | 90(35.7) | 0.08 | 0.72(.50-1.04) |
| # p value less than 0.05 is considered as statistically significant | | | | |
